# Vitamin B_6_: a scoping review for Nordic Nutrition Recommendations 2023

**DOI:** 10.29219/fnr.v67.10259

**Published:** 2023-12-19

**Authors:** Anne-Lise Bjørke-Monsen, Per Magne Ueland

**Affiliations:** 1 Department of Medical Biochemistry and Pharmacology, Haukeland University Hospital, 5021 Bergen, Norway; 2 Department of Clinical Science, University of Bergen, Bergen, Norway

**Keywords:** vitamin *B*_6_, PLP, nutrition recommendations

## Abstract

Pyridoxal 5´-phosphate (PLP) is the main form of vitamin B_6_ in animal tissue and functions as a coenzyme for more than 160 different enzymatic reactions in the metabolism of amino acids, carbohydrates, lipids, and neurotransmitters. Estimated dietary intake of vitamin B_6_ and plasma PLP values differ a lot between studies, something which may be due to variable use of supplements, variations in dietary assessment and analytical methods. These factors make it difficult to achieve precise data for setting a correct recommended intake of vitamin B_6_. In addition, a plasma PLP concentration of 30 nmol/L is considered to be sufficient and the current recommendations for vitamin B_6_ intake is based on this concept. However, the metabolic marker for vitamin B_6_ status, HK ratio (HKr), starts to increase already when plasma PLP falls below 100 nmol/L and increases more steeply below 50 nmol/L, indicating biochemical deficiency. Consequently, a plasma PLP concentration of 30 nmol/L, may be too low as a marker for an adequate vitamin B_6_ status.

## Popular scientific summary

- Vitamin B_6_ plays an essential role as a coenzyme for multiple biochemical reactions in the body.
- Plasma levels of pyridoxal 5´-phosphate (PLP) is a biomarker of vitamin B_6_ intake and the most commonly used basis for dietary recommendations.
- Major dietary sources of vitamin B_6_ in the Nordic population are fish, meat, offal, potatoes, bread, cereals, milk, and dairy products.
- The classic symptoms of vitamin B_6_ deficiency are microcytic anaemia, depression, and confusion.
- Inflammation-related diseases, such as cardiovascular disease, diabetes, rheumatoid arthritis, and inflammatory bowel disease, has been associated with low vitamin B_6_ status.

The aim of this scoping review is to describe the evidence for the role of vitamin B_6_ for health-related outcomes, in addition to the various metabolic and methodological instruments one need to consider for setting and updating dietary reference values (DRVs) in the Nordic Nutrition Recommendations 2023 (Box 1).

Box 1 The Nordic Nutrition Recommendations (NNR) 2023 project

- This article is one of many scoping reviews commissioned as part of the Nordic Nutrition Recommendations 2023 (NNR2023) project (11).
- The articles are included in the extended NNR2023 report but, for transparency, these scoping reviews are also published in Food & Nutrition Research.
- The scoping reviews have been peer reviewed by independent experts in the research field according to the standard procedures of the journal.
- The scoping reviews have also been subjected to public consultations (see report to be published by the NNR2023 project).
- The NNR2023 committee has served as the editorial board.
- While these articles are a main fundament, the NNR2023 committee has the sole responsibility for setting dietary reference values in the NNR2023 project…

Vitamin B_6_ is the common term for pyridoxal, pyridoxine and pyridoxamine and their 5′-phosphate forms, of which all have vitamin activity. Pyridoxal 5´-phosphate (PLP) is the main form of vitamin B_6_ in animal tissue and makes up 70–90% of the total vitamin B_6_ in plasma. PLP is a coenzyme for more than 160 different enzymatic reactions in the metabolism of amino acids, carbohydrates, lipids, and neurotransmitters (1).

Vitamin B_6_ status can be assessed using a variety of biochemical indicators, of which the plasma PLP level is the most commonly used (2). Metabolically, PLP deficiency increases the ratio between 3-hydroxykynurenine and the sum of kynurenic acid (KA) + anthranilic acid (AA) + xanthurenic acid (XA) + hydroxyanthranilic acid (HAA), termed HK ratio (HKr), a proposed marker of vitamin B_6_ deficiency (1, 3).

Low vitamin B_6_ status, based on plasma PLP concentrations, has been identified in diseases associated with low-grade and overt inflammation, including cardiovascular disease, diabetes, rheumatoid arthritis, and inflammatory bowel disease (4).

Classic symptoms of vitamin B_6_ deficiency are microcytic anaemia, depression, and confusion, most of which are related to the role of vitamin B_6_ as a coenzyme in haemoglobin and neurotransmitter biosynthesis (5). Poor vitamin B_6_ status appears to decrease the probability of conception and to contribute to the risk of early pregnancy loss (6).

Major sources of vitamin B_6_ in the Nordic diets are fish, meat, offal, potatoes, bread, cereals, milk, and dairy products. The bioavailability of vitamin B_6_ is estimated to be >75% from food in a mixed Western diet and >90% from supplements (5). Both estimated dietary intake of vitamin B_6_ and plasma PLP values differ a lot between studies (4, 7). The reported variation in intake for vitamin B_6_ is mainly due to variable use of supplements, but is also related to the different dietary assessment methods. The high variability of B_6_ vitamers and their low concentrations in food products are known to cause difficulties in analysis, something which may affect the estimated intake of B_6_ from foods (8). In addition, non-optimal sample handling or storage of blood samples will convert PLP to pyridoxal, thereby reducing PLP concentrations (9). These factors make it difficult to achieve precise data for setting a correct recommended intake of vitamin B_6_.

Current recommendations for vitamin B_6_ intake are based on the concept that a plasma PLP concentration of 30 nmol/L is considered to ensure a sufficient vitamin B_6_ status. However, the metabolic marker KHr starts to increase when plasma PLP falls below 100 nmol/L and increases more steeply below 50 nmol/L (10). Based on this, a plasma PLP concentration of 30 nmol/L may be too low to ensure an optimal vitamin B_6_ status.

## Methods

This review follows the protocol developed within the NNR2023 project (11). The sources of evidence used in the scoping review follow the eligibility criteria described previously (2).

General literature search was performed by the NNR committee on November 1st, 2019 in MEDLINE with a search string: (vitamin b6[MeSH Terms]) OR pyridoxine[MeSH Terms] OR “vitamin b6”[Title] OR pyridoxine[Title]) AND review[Publication Type]) AND (“2011”[Date - Publication] : “3000”[Date - Publication]) AND humans[Filter]. The number of hits was 149. Based on the title, four articles were retrieved, of which two were considered as relevant based on the full articles. Of these two, none were qualified systematic reviews. No *de novo* systematic review was conducted on vitamin B_6_. We also identified relevant literature for this scoping review via ‘snowballing’/citation chasing in February 2022 and six relevant systematic reviews and meta-analyses were identified (12, 13, 14, 15, 16, 17).

## Physiology

Vitamin B_6_ is the common term for pyridoxal, pyridoxine and pyridoxamine and their 5′-phosphate forms, of which all have vitamin activity. The bioavailability of vitamin B_6_ in foods varies and depends on the chemical form of the vitamin (18). Studies indicate that pyridoxal and pyridoxamine raise the PLP concentration by about 10% less than pyridoxine. A glycosylated form of pyridoxine, pyridoxine-glucoside, comprises 5–70% of the total vitamin B_6_ in selected fruits and vegetables, but are not found in animal-derived foods including meats, human milk, and cow’s milk. This conjugate has been shown to exhibit incomplete metabolic utilisation as vitamin B_6_ (19). The content of pyridoxine-glucoside in a mixed American diet has been estimated to be about 15% of the total vitamin B_6_ content (20). While the bioavailability of B_6_ from animal products is quite high, reaching 100% for many foods, the presence of fibre in plant food reduces the bioavailability by 5–10% and the presence of pyridoxine glucoside reduces it by 75–80%. This glucoside is found in a variety of plant foods, with the highest content occurring in the crucifers, and PLP concentrations in vegetarians may be adversely affected by dietary intake of the naturally occurring pyridoxine glucoside (21). The absorption of the different vitamin B_6_ vitamers has been thought to occur via a passive process in the gut; however, a transport mechanism, that is, SLC19A2 and SLC19A3, known as thiamine transporter 1 (THTR1) and THTR2(22, 23), has recently been identified (24).

The body stores of vitamin B_6_ have been estimated to be approximately 1000 µmol (170 mg), of which 80–90% is found in the muscles, where PLP has a covalent bond to glycogen phosphorylase (25). Phosphorylation of PLP enzymes play important roles in mediating diverse cellular functions, but it is not fully understood (26). The turnover of the vitamin is relatively fast in plasma with a half-life of 25–33 days for PLP, but much slower in muscle tissue (27).

**Fig. 1:**
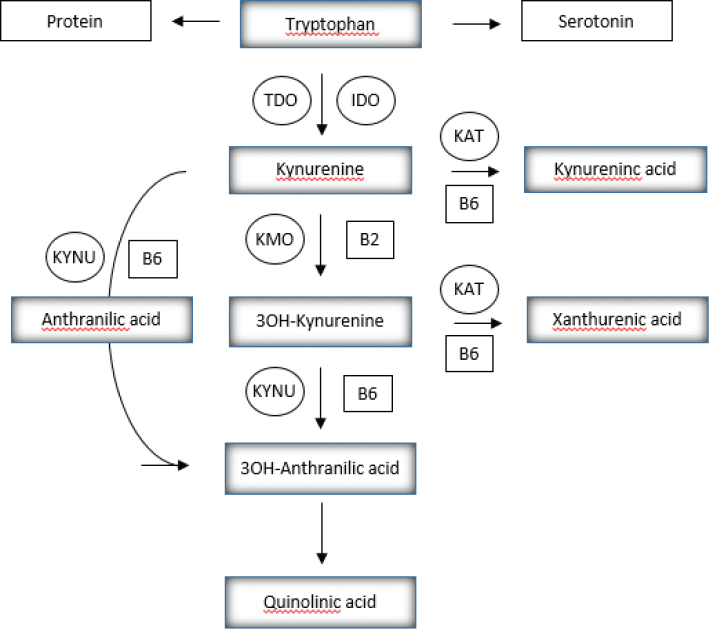
PLP is a cofactor in the conversion of kynurenine to kynurenic acid by kynurenine aminotransferase (KAT), and to anthranilic acid by kynureninase (KYNU), as well as in the conversion of 3-hydroxykynurenine to either xanthurenic acid by KAT or to 3-hydroxyanthranilic acid by KYNU. Tryptophan 2,3-dioxygenase (TDO); Indoleamine 2,3-dioxygenase (IDO); kynurenine 3-monooxygenase (KMO).

PLP is a cofactor for more than 160 different enzymes, which have an important role in the metabolism of amino acids, including biosynthesis and catabolism of neurotransmitters, such as dopamine, serotonin, glycine, glutamate, and γ-aminobutyric acid (GABA), and also organic acids, glucose, sphingolipids, and fatty acids (1). PLP is a cofactor for cystathionine beta-synthase (CBS) enzyme and four enzymes in the catabolism of tryptophan through the kynurenine pathway (Fig. 1).

### Pregnancy

Plasma PLP decrease throughout pregnancy and increase postpartum, while the metabolic marker HKr increase from week 18 to 6 weeks postpartum, indicating maternal vitamin B_6_ insufficiency during this period (28). In a study published in 1992, plasma PLP was substantially higher in the new-born, while the maternal plasma PLP concentration was 22.2 nmol/L, the concentration in venous cord plasma was 112.1 nmol/L (29), indicating that pregnant women have an increased requirement for vitamin B_6_.

### Breast milk

A literature review published in 2012 concluded that the predominant form of vitamin B_6_ in breast milk is pyridoxal (75%), with smaller amounts of PLP (9%), pyridoxamine, and pyridoxine. The concentration of vitamin B_6_ in breast milk is low during the first 1–2 weeks post-partum, but increase gradually with the progression of lactation (30). A significant positive association has been reported between the age of the infant in months and total vitamin B_6_ concentration in breast milk (31).

Maternal supplementation with pyridoxine produces a rapid increase in milk concentrations of all the different vitamin B_6_ vitamers (32). Breast milk vitamin B_6_ concentrations are reported to increase with maternal supplementation >2.5 mg/day (33). Mothers with vitamin B_6_ intake higher than the median value of 2.90 mg/day had a significantly higher median pyridoxal level in their breast milk than did the mothers with intakes below the median value (34). Based on small studies published more than 20 years ago (20, 35), the mean concentration of vitamin B_6_ in mature breast milk was rounded up to an average of 130 μg/L (36).

### Infants

Foetal plasma PLP concentrations from mid-pregnancy and at term are significantly higher than in the mother, suggesting foetal sequestration of the vitamin (29, 37). The vitamin B_6_ intake of breastfeeding mothers has been shown to be a strong predictor of infant status (38). However, in a recently published study, infant median plasma PLP decreased with months of exclusive breastfeeding, despite correcting for maternal vitamin B_6_ status (28).

### Infants born premature or with a low birth weight

Infants born premature or with a low birth weight have lower stores of all micronutrients and have a risk of developing deficiency during the first months of life. Infants with a birth weight between 2,500 – 3,000 g, who were exclusively breast fed for >1 month had lower plasma PLP concentrations at 6 weeks, 4 and 6 months compared with infants who were formula-fed (39). In formula-fed infants median plasma PLP decreased from 274 (IQR: 201, 337) nmol/L at 6 weeks to 184 (123, 278) nmol/L at 6 months, whereas in exclusively breastfed infants, median plasma PLP increased from 6 weeks median 79 (42, 132) nmol/L to median 122 (92, 162) nmol/L at 6 months of age (39).

### Older children

In a study from the UK published in 2012, plasma PLP ranged from 46 to 321 nmol/L in healthy children aged 4.3 to 16 years (40). Comparing two British national surveys in subjects aged 4–18 years (*n* = 1,006) or 65 years and over (*n* = 919), geometric mean plasma PLP concentration was significantly higher in children than in older adults (56.5 vs. 34.0 nmol/L, *P* < 0.001) (41).

## Assessment of nutrient status

Vitamin B_6_ status can be assessed using a variety of biochemical indicators, which are categorised as direct biomarkers and as functional biomarkers. Direct biomarkers measure B_6_ vitamers in plasma, serum, urine and erythrocytes, and among these plasma PLP is most commonly used (2). PLP makes up 70–90% of the total vitamin B_6_ in plasma, and this level reflects both the tissue stores and intake of vitamin B_6_. PLP levels might also be affected by factors independent of the dietary intake, such as age, pregnancy, inflammation and physical exercise (42).

**Fig. 2:**
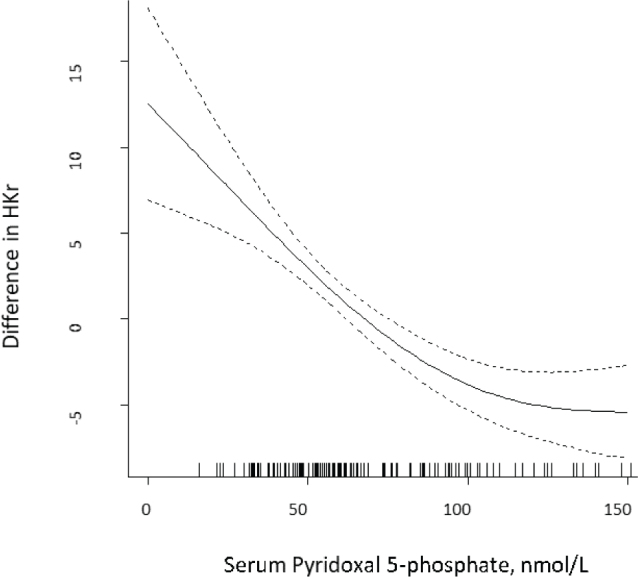
Plasma pyridoxal 5´-phosphate in women of fertile age (*n* = 158) in relation to HKr by generalised additive model (GAM). The values on the y-axes are given as difference from the respective mean values (unpublished data). The dotted lines represent the 95% confidence intervals of the GAM estimate.

Functional biomarkers include erythrocyte transaminase activities and, plasma levels of metabolites involved in PLP-dependent reactions, such as the kynurenine pathway, one-carbon metabolism, transsulphuration (cystathionine), and glycine decarboxylation (serine and glycine) (1). Vitamin B_6_ status is best assessed by using a combination of biomarkers because of the influence of potential confounders, such as inflammation, alkaline phosphatase activity, low serum albumin, renal function, and inorganic phosphate. Ratios between substrate-products pairs have recently been investigated as a strategy to attenuate such influence. These efforts have provided promising new markers such as the PAr index, the 3-hydroxykynurenine:xanthurenic acid ratio, and the oxoglutarate:glutamate ratio (1). PLP deficiency may increase the ratio between 3-hydroxykynurenine/xanthurenic acid, termed the HK/XA ratio, and also the ratio between 3-hydroxykynurenine (HK) / the sum of kynurenic acid (KA) + anthranilic acid (AA) + xanthurenic acid (XA) + hydroxyanthranilic acid (HAA), termed HK ratio (HKr), two proposed markers of vitamin B_6_ status (1, 3), none have been related to clinical symptoms of deficiency. In adults, both ratios increase with decreasing plasma PLP levels, starting from plasma PLP levels ~ 100 nmol/L, with a steeper increase below 50 nmol/L, indicating that below these plasma PLP levels, intracellular PLP content becomes a rate-limiting factor for the metabolic flux across the kynurenine pathway (3, 10) (Fig. 2). Studies on changes in patterns of plasma metabolites, including organic acids and amino acids, in people with plasma PLP less than 30–50 nmol/L suggest that these PLP concentrations should be viewed as inadequate (28, 43, 44). In pregnancy week 28 a sharp increase in HKr was seen at plasma PLP<30 nmol/L (28).

Moderate vitamin B_6_ insufficiency weakly increases plasma tHcy level because of the PLP-dependence of the two enzymes in the trans-sulphuration pathway. Plasma tHcy is however more strongly influenced by folate and vitamin B_12_ status (45). Severe B_6_ deficiency due to inadequate diet or use of decarboxylase inhibitors (like Sinemet^®^), causes plasma tHcy concentrations in the range observed in homocystinuria (>100 µmol/L), indicating that the transulphuration pathway may be metabolically preferred in patients with moderate vitamin B_6_ deficiency (46).

Low vitamin B_6_ status, based on plasma PLP concentrations, has been identified in diseases associated with low-grade or overt inflammation, including cardiovascular disease, diabetes, rheumatoid arthritis, and inflammatory bowel disease (4). Inflammatory conditions and increased concentration of inflammatory markers in the circulation are associated with reduced plasma PLP concentration. The effect of inflammation on vitamin B_6_ status may occur by sequestration of PLP in inflammatory tissues (47) and/or by increased catabolism (42).

## Dietary intake in Nordic and Baltic countries

All organisms are dependent on vitamin B_6_, but only microorganisms and plants are able to synthesise it de novo, and humans rely on uptake of the vitamin from food. Major sources of vitamin B_6_ in the Nordic diets are fish, meat, offal, potatoes, bread, cereals, milk and dairy products. The bioavailability of vitamin B_6_ in animal foods are considered to be approximately 50%, whereas the bioavailability in plant-based foods varies considerably, ranging from 0 to 80% (48). Isolated B_6_ deficiency is rare and usually found in association with deficiencies of other B vitamins such as folate and B_12_ (49). A recent systematic review based on data from 27 studies showed that average vitamin B_6_ intake tended to be higher in vegans (2.81 mg/d) compared with vegetarians and meat-eaters (1.82 mg/d), irrespective of whether studies assessed intake from supplements (50). As plant food contains various amounts of pyridoxine-glucoside, which reduces the bioavailabilty (19), the estimated intake of pyridoxine might not be reflected in PLP status. However, the systematic review, based on eight studies, found that average vitamin B_6_ levels were similar for meat-eaters, vegetarians, and vegans (50).

Reported intake ranges for vitamin B_6_ vary considerably in the literature, mainly due to the use of supplements and, to some extent, to the dietary assessment method used. Although reported intakes from dietary sources seem to be relatively similar in Europe and the US, total intakes in several US studies are higher due to the common use of supplements. Inclusion of supplements introduces uncertainties in the interpretation of results because most supplements contain a variety of B-vitamins and other micronutrients. Alcohol dependence, obesity, pregnancy, chronic renal failure, and malabsorption are associated with an increased risk of B vitamin deficiency (1). Apart from decarboxylase inhibitors, isoniazid (used to treat tuberculosis) and penicillamine (used to treat Wilson’s disease, cystinuria, and rheumatoid arthritis) are reported to induce B_6_ deficiency (51).

A varied omnivore diet is reported to provide a daily amount of 6–9 mg vitamin B_6_, considered to be adequate for adults (49). However, updated data on nutrient intakes and food consumption in the adult population from the Nordic and Baltic countries from a recently published article report much lower intakes (52). Average dietary intake in the Nordic countries according to national dietary surveys are reported to ranges from 1.4 to 1.8 mg/day in adult women and from 1.8 to 2.3 mg/day in adult men. Mean B_6_ intake in Estonia was somewhat lower (mean 1.2 mg/day in women and 1.5 mg/day in men) (52).

In children, B_6_ intake ranges from 1.0 to 1.2 mg/day. It is of interest that Swedish children have higher estimated intake; for age-group 12 years: 1.6 and 1.7 mg/day, 15 years: 1.8 and 2.2 mg/day and for age-group 18 years: 2.1 and 2.8 mg/day, for girls and boys, respectively (52).

Based on data from 13 surveys in nine countries of the European Union, average total vitamin B_6_ intake ranges across countries from 0.4 to 0.8 mg/day in infants, from 0.9 to 1.3 mg/day in children aged 1 to <3 years, from 1 to 1.6 mg/day in children aged 3 to <10 years, and from 1.5 to 2.3 mg/day in children aged 11 to <18 years. Average total vitamin B_6_ intake ranges between 1.4 and 3.1 mg/day in adults (36).

## Health outcomes relevant for Nordic and Baltic countries

### Vitamin B_6_-dependent epilepsy in neonates

Pyridoxine-dependent epilepsy (PDE) is a rare neurometabolic disease with a prevalence of 1/20,000 – 1/783,000 live births. PDE is characterised by recurrent intractable seizures in the prenatal, neonatal and postnatal period that are resistant to anti-epileptic drugs (AEDs), but that are responsive to pharmacological dosages of pyridoxine. PDE is caused by mutations in the *ALDH7A1* gene (5q31) that encodes alpha-aminoadipic semialdehyde dehydrogenase (antiquitin), a multifunctional enzyme which, among other functions, is involved in the catabolism of lysine (53).

Pyridoxal-5′-phosphate-dependent epilepsy is caused by autosomal recessive mutations in the pyridox(am)ine 5′-phosphate oxidase (PNPO) gene encoding for pyridox(am)ine 5′-phosphate oxidase, an enzyme needed for the conversion of pyridoxine and pyridoxamine into PLP. In contrast to PDE, patients with PNPO deficiency suffer from systemic PLP deficiency, which may explain the broader organ involvement and very high mortality in untreated patients. Diagnosis of PNPO deficiency is established by measurement of low PLP levels in plasma and CSF (54).

### Deficiency

Prolonged vitamin B_6_ deficiency is reported to cause a painful axonal peripheral neuropathy that leads to weakness, decreased reflexes, sensory loss and ataxia, particularly in the lower limbs (55). However, a recent systematic review and meta-analysis of associations between neuropathy and B vitamins showed an association of neuropathy pain and low vitamin B_12_ and elevation of methylmalonic acid, an indicator of B_12_ deficiency, but no significant relationship with vitamin B_6_ status (16). Some evidence exists for reduction of pain in diabetic neuropathy during supplementation with a supplement containing sources of folate, vitamin B_12_ and vitamin B_6_, but there was no significant effect on vibration perception threshold, which was the primary outcome measure of the randomized controlled trial (RCT) (56). Seizures, migraine, cognitive decline and depression have been linked to vitamin B_6_ deficiency (57).

Vitamin B_6_ deficiency is reported to exacerbate anorexia, due to its effect on serotonin metabolism and appetite (58). A classic symptom of vitamin B_6_ deficiency is microcytic anaemia (5). The rate limiting enzyme in haeme biosynthesis is the PLP dependent 5-ALA synthase (5-ALAS). Vitamin B_6_ deficiency will reduce the activity of 5-ALAS activity and may thus cause anaemia (59, 60).

Long term high dose combined use of L-dopa and carboxylase inhibitors for Parkinson’s disease may cause functional vitamin B_6_ deficiency (47).

A prospective observational study in China found a decreased probability of conception and an increased risk of early pregnancy loss in women in the lowest quartile of vitamin B_6_ level and in women with vitamin B_6_ deficiency (6). In animal studies severe maternal vitamin B_6_ deficiency has been associated with lower body weight, skeletal defects, convulsions and impaired neuromotor development in the offspring (61, 62), but no such associations have been documented in humans (5).

### Upper intake levels and toxicity

Adverse effects of high vitamin B_6_ intakes have been observed at intakes above 50 mg/d consumed for prolonged periods of months to years. Symptoms include minor neurological symptoms and, at higher levels of 500 mg/d or more, neurotoxicity (63). The EU Scientific Committee on Food concluded in 2000 and 2012 that adverse effects are unlikely to occur at doses below 100 mg/d and proposed an upper safe intake level (UL) for adults of 25 mg/d (63).

However, in 2023 this UL was reduced to 12 mg/day vitamin B_6_ for adults (including pregnant and lactating women). ULs for infants and children are derived from the UL for adults using allometric scaling: 2.2–2.5 mg/day (4–11 months), 3.2–4.5 mg/day (1–6 years), 6.1–10.7 mg/day (7–17 years). Based on available intake data, EU populations are unlikely to exceed ULs, except for regular users of food supplements containing high doses of vitamin B_6_ (64).

### Cardiovascular disease

A systematic review and meta-analysis published in 2013 reported a slightly decreased risk of major cardiovascular events with low dose vitamin B_6_ supplementation. However, this effect was only seen in controlled trials in which the supplements were supplied by the pharmaceutical industry. The conclusion was that there is no evidence to support the use of vitamin and antioxidant supplements for prevention of cardiovascular disease (15).

### Cancer

A systematic review of both observational and intervention studies concluded that a high intake of dietary (food only) vitamin B_6_ was statistically significantly associated with lower risk of all cancers (relative risk [RR] = 0.78, 95% confidence interval [CI] = 0.73 to 0.84) and specific tumours, with special regard to gastrointestinal carcinomas (RR = 0.68, 95% CI = 0.61 to 0.75) (14). A dose-response meta-analysis showed a statistically significant inverse dose-response relationship with a 6% risk reduction per milligram of vitamin daily intake and any type of cancer (RR = 0.94, 95% CI = 0.92 to 0.96). An inverse association was also observed between high PLP levels and the risk of all cancers (RR = 0.66, 95% CI = 0.58 to 0.76). Dose-response meta-analysis demonstrated a statistically significant inverse association between vitamin blood levels and all tumour sites (30% risk reduction per 100 nmol/L of blood PLP (RR = 0.70, 95%, CI = 0.65 to 0.76). For single tumour sites, the most consistent results being those for gastrointestinal tumours (RR = 0.56, 95% CI = 0.48 to 0.65). There was a statistically significant inverse linear relationship between cancer risk and both vitamin B_6_ dietary intake and PLP levels. When total (food and supplements) intake was considered, the associations were weaker or null, suggesting that vitamin B_6_ intake might also be an indicator of other dietary protective micronutrients (14).

### Cognitive function

A systematic review published in 2021 found only small, cross-sectional study on the association between vitamin B_6_ in breast milk and neurodevelopment in neonates (13). However, in one small study, infant scores on habituation and autonomic stability subscales of the Brazelton Neonatal Behavioural Assessment Scale were positively correlated with milk pyridoxal values at 8–11 days postpartum (*n* = 25) (34). A recent systematic review and meta-analysis of vitamins B_6_, B_12_ and folate in cognitive function in community dwelling older adults reported that vitamin B_6_ status was not associated with risk of cognitive decline or dementia (17). The same result has been reported previously (57) and is supported by prospective cohort studies that found no statistically significant associations between risk of dementia or Alzheimer’s disease and total intake of dietary vitamin B_6_ and supplements (65, 66) or between cognitive function and plasma PLP (67).

## Requirement and recommended intakes

In NNR 2004 it was noticed that current literature was unclear regarding whether or not it would be beneficial for the RI to be based on achieving plasma PLP of 30 nmol/L. Recommended vitamin B_6_ intake in 2012 (68) was based on the results from depletion-repletion studies with controlled intakes of vitamin B_6_ (expressed as free pyridoxine) showing that PLP levels above 20 nmol/L could be reached at intakes of 0.6–1.0 mg/d or around 0.01 mg/g dietary protein (62, 63, 65–69). The estimated average requirement (AR) of vitamin B_6_ for adult men and women was set at 0.013 mg/g dietary protein. However, a recent publication shows that higher plasma PLP concentrations are associated with a better metabolic status (28). A plasma PLP concentration in the range of 50 – 100 nmol/L seems to ensure an optimal vitamin B_6_ status for never-pregnant women, whereas a plasma PLP > 30 nmol/L in pregnancy week 28 ensures an adequate vitamin B_6_ status during pregnancy and lactation (28).

In the US NHANES study in more than 6,000 individuals older than 1 year, after multivariate adjustment, plasma PLP increased by about 12 nmol/L per 1 mg increase in daily vitamin B_6_ intake (*P* < 0.001) (70). Among US individuals aged 13–54 years, mean plasma PLP was 40 nmol/L with a vitamin B_6_ intake <2 mg/day, PLP was 49 nmol/L with a B_6_ intake 2–2.9 mg/day and PLP was 54 nmol/L with a B_6_ intake 3–4.9 mg/day and PLP was 108 nmol/L with a B_6_ intake ≥ 5 mg/day (70). Increasing vitamin B_6_ intake is associated with higher plasma PLP levels in US adults with mean age of 61 ± 9 years. Median plasma PLP (IQR) was 35 (34, 36) nmol/L with a mean B_6_ intake of 2.7 mg/d, median PLP was 69 (70) nmol/L with a mean B_6_ intake of 5.2 mg/d, and median PLP was 177 (173, 181) nmol/L with a mean B_6_ intake of 18.6 mg/d (4).

In another study, young women consumed a controlled diet containing four levels of vitamin B_6_, providing 1.0 mg vitamin B_6_ per day for 1 week, followed by 1.5, 2.1 and 2.7 mg vitamin B_6_ per day; each study period lasted 2 weeks (7). Baseline vitamin B_6_ intake was estimated to be 1.4 mg/d. The baseline mean plasma PLP concentrations of 46.6 (SD: 13.9) nmol/L was reduced to 29.7 (SD: 6.7) nmol/L after 1 week of 1.0 mg vitamin B_6_ per day, plasma PLP increased to mean 35.2 (SD: 6.0) nmol/L after 2 weeks intake of 1.5 mg vitamin B_6_ per day, increased further to mean 43.7 (SD: 7.2) nmol/L after 2 weeks of 2.1 mg vitamin B_6_ per day, and finally to mean 56.1 (SD: 13.2) nmol/L after 2 weeks with 2.7 mg vitamin B_6_ per day (7). These data indicate that the estimated baseline intake of 1.4 mg per day may have been regarded too low, as a plasma PLP concentration of mean 46.6 (SD: 13.9) nmol/L coincided with an intake of 2.1 mg per day. With a mean vitamin B_6_ intake of 2.69 (standard deviation [SD]: 1.30) mg/d, Puerto Rican adults, aged 45–75 years had a geometric mean plasma PLP of 44.3 nmol/L and 28% had a PLP level <30 nmol/L, indicative of marginal insufficiency (71).

EFSA’s AR and population reference intake (PRI) values were based on a vitamin B_6_ intake yielding a plasma PLP of 30 nmol/L, considered to be a sufficient vitamin B_6_ status (36). PRIs were derived for adults and children from ARs, assuming a coefficient of variation (CV) of 10%. For adult women the AR and PRI were set at 1.3 and 1.6 mg/day and for men 1.5 and 1.7 mg/day, respectively. The Nutrition Societies of Germany, Austria, and Switzerland published updated recommendations in 2020 (72). The recommended AR for vitamin B_6_ to ensure a plasma PLP concentration of ≥30 nmol/L is 1.2 mg/day for adult females and for males 1.3 mg/day. The corresponding RIs are 1.4 and 1.6 mg/day, independent of age.

### Pregnancy and lactation

Mean dietary vitamin B_6_ intake from diet and supplements among pregnant Norwegian women in the MoBa study, based on self-reporting in pregnancy week 17–24, was mean 4.6 (SD: 11) mg/d, of which supplements constituted mean 3.1 (SD: 11) mg/day (73). Approximately one- third of the women had an intake below recommended 1.8 mg/day (NNR 2012) (73). Forty percent of the women reported taking vitamin B_6_ in addition to folate supplements, and in supplements users, median plasma PLP was 29.3 (IQR: 21.9–42.0), compared with 24.1 (18.6–30.4) nmol/L in non-users in pregnancy week 18 (74). These PLP concentration may however be falsely low due to non-optimal preanalytical handling in the MoBa study, something which is known to reduce PLP concentrations (9).

Plasma PLP concentrations are reported to decrease during pregnancy and increase postpartum, while the metabolic markers HKr increase from week 18 to 6 weeks postpartum, indicating maternal vitamin B_6_ insufficiency during this period (28). There is, however, no agreement if this merely reflects physiological changes or maternal deficiency. Accordingly, the recommended additional B_6_ intake varies from 0 to 0.7 mg/day, and total recommended intake varies from 1.2 to 2.0 mg/day (5, 36, 72).

The basic requirement for vitamin B_6_ is increased for pregnant women, especially during the last trimester, to cover the extra needs of the foetus. During the last two trimesters of pregnancy and during lactation, an additional intake of 0.2 mg/d and 0.3 mg/d, respectively, is recommended (68). EFSA derived a PRIs of 1.8 for pregnant women (36).

For lactating women, an increased intake is necessary to cover the needs for vitamin B_6_ in breast milk. Based on an average production of 0.8 L breast milk per day (74), and a mean B_6_ concentration of 0.130 mg/L, the mother will loose an estimated mean of 0.1 mg vitamin B_6_ per day during the first 6 post-partum months (36). Assuming a bioavailability of vitamin B_6_ of 75%, a mean vitamin B_6_ intake of 0.133 mg/day is required to balance the amount of vitamin B_6_ secreted in milk for exclusively breastfeeding women during the first 6 months of lactation. This intake, added to the AR of non-lactating women (1.3 mg/day), results in an AR of 1.4 mg/day vitamin B_6_. Assuming a CV of 10%, a PRI of 1.7 mg/day vitamin B_6_ is derived for exclusively breastfeeding women by EFSA (36). The Nutrition Societies of Germany, Austria, and Switzerland set an AR of 1.3 mg/day in the first trimester and 1.5 mg/day in the second and third trimesters; the RI is 1.5 mg/day in the first trimester and 1.8 mg/day in the second and third trimesters. For lactating women, the AR is 1.3 mg/day and the RI is 1.6 mg/day (72).

### Infants and older children

For infants and older children, the NNR 2012 reference intakes were based on the same value as for adults due to a lack of scientific data to suggest otherwise. In American children <13 years mean plasma PLP was 36 nmol/L with a vitamin B_6_ intake <2 mg/day, PLP was 40 nmol/L with a B_6_ intake 2–2.9 mg/day and PLP was 54 nmol/L with a B_6_ intake 3–4.9 mg/day (70). EFSA set an adequate intake (AI) at 0.3 mg/ day for infants aged 7–11 months,. For children aged 1–14 years, ARs ranged between 0.5 and 1.2 mg/day. For children aged 15–17 years, the Panel derived the same ARs as for adults. PRIs for children aged 1–17 years ranged between 0.6 and 1.7 mg/day. The Nutrition Societies of Germany, Austria, and Switzerland estimated value for infants is 0.1 and 0.3 mg/day, depending on age. The AR of vitamin B_6_ for children and adolescents ranges between 0.5 and 1.5 mg/ day, and the RI is between 0.6 and 1.6 mg/day (72).

Table 1 shows that the estimated vitamin B_6_ intake and the corresponding plasma PLP values vary a lot. This may be due to both preanalytical and methodological issues. The high variability of B_6_ vitamers and their low concentrations in food products are known to cause difficulties in analysis, something which may affect estimated intake of B_6_ from foods (8). Additionally, non-optimal sample handling or storage of blood samples will convert PLP to pyridoxal, thereby reducing PLP concentrations (9). These factors make it difficult to achieve precise data for setting a correct recommended intake. There are also few recent studies, particularly on pregnant and lactating women and infants, something which also makes it difficult to find the correct recommendation.

**Table 1:** Estimated vitamin B_6_ intake and associated plasma PLP concentrations in adults

	B_6_ intake, mg/day					
	1.0	<2	2–2.9	3–4.9	≥5	Ref.
**Plasma PLP, nmol/L**		40	49	54	108	(71)
			35		69	(4)
	29.7	35.2–46.6	43.7–56.1			(7)
